# Prevention and early treatment of the long-term physical effects of COVID-19 in adults: design of a randomised controlled trial of resistance exercise—CISCO-21

**DOI:** 10.1186/s13063-022-06632-y

**Published:** 2022-08-15

**Authors:** A. Morrow, Stuart R. Gray, H. K. Bayes, R. Sykes, E. McGarry, D. Anderson, D. Boiskin, C. Burke, J. G. F. Cleland, C. Goodyear, T. Ibbotson, C. C. Lang, McConnachie, F. Mair, K. Mangion, M. Patel, N. Sattar, D. Taggart, R. Taylor, S. Dawkes, C. Berry

**Affiliations:** 1 Institute of Cardiovascular and Medical Sciences, University of Glasgow, Glasgow, UK; 2 Glasgow Royal Infirmary, NHS Greater Glasgow and Clyde, Glasgow, UK; 3 Queen Elizabeth University Hospital, NHS Greater Glasgow and Clyde, Glasgow, UK; 4 Robertson Centre for Biostatistics, Institute of Health and Wellbeing, University of Glasgow, Glasgow, UK; 5 Institute of Inflammation, Infection and Immunity, University of Glasgow, Glasgow, UK; 6 General Practice and Primary Care, Institute of Health and Wellbeing, University of Glasgow, Glasgow, UK; 7 School of Medicine, University of Dundee, Dundee, UK; 8 University Hospital Wishaw, NHS Lanarkshire, Wishaw, UK; 9 NHS Project Management Unit, NHS Greater Glasgow and Clyde, Glasgow, UK; 10 School for Nursing Midwifery and Paramedic Practice, Robert Gordon University, Aberdeen, UK

**Keywords:** Resistance exercise, Long COVID, SARS-CoV-2, COVID-19, Rehabilitation, Randomised controlled trial

## Abstract

**Background:**

Coronavirus disease-19 (COVID-19) infection causes persistent health problems such as breathlessness, chest pain and fatigue, and therapies for the prevention and early treatment of post-COVID-19 syndromes are needed. Accordingly, we are investigating the effect of a resistance exercise intervention on exercise capacity and health status following COVID-19 infection.

**Methods:**

A two-arm randomised, controlled clinical trial including 220 adults with a diagnosis of COVID-19 in the preceding 6 months. Participants will be classified according to clinical presentation: Group A, not hospitalised due to COVID but persisting symptoms for at least 4 weeks leading to medical review; Group B, discharged after an admission for COVID and with persistent symptoms for at least 4 weeks; or Group C, convalescing in hospital after an admission for COVID.

Participants will be randomised to usual care or usual care plus a personalised and pragmatic resistance exercise intervention for 12 weeks. The primary outcome is the incremental shuttle walks test (ISWT) 3 months after randomisation with secondary outcomes including spirometry, grip strength, short performance physical battery (SPPB), frailty status, contacts with healthcare professionals, hospitalisation and questionnaires assessing health-related quality of life, physical activity, fatigue and dyspnoea.

**Discussion:**

Ethical approval has been granted by the National Health Service (NHS) West of Scotland Research Ethics Committee (REC) (reference: GN20CA537) and recruitment is ongoing. Trial findings will be disseminated through patient and public forums, scientific conferences and journals.

**Trial registration:**

ClinicialTrials.gov NCT04900961. Prospectively registered on 25 May 2021

**Supplementary Information:**

The online version contains supplementary material available at 10.1186/s13063-022-06632-y.

## Administrative information

Note: the numbers in brackets in this protocol refer to SPIRIT checklist item numbers. The order of the items has been modified to group similar items (see http://www.equator-network.org/reporting-guidelines/spirit-2013-statement-defining-standard-protocol-items-for-clinical-trials/).

**Table:** 

Title {1}	Protocol for prevention and early treatment of the long-term physical effects of COVID-19 in adults: a randomised controlled trial of resistance exercise—CISCO-21
Trial registration {2a and 2b}.	Prospectively registered on 25^th^ May 2021, trial registration clinicialtrials.gov ID NCT04900961 https://clinicaltrials.gov/ct2/show/NCT04900961
Protocol version {3}	Version 2.0 31.01.2022
Funding {4}	Chief Scientist Office Scotland and the University of Glasgow British Heart Foundation Centre of Research Excellence (RE/18/634217) – the funders had no role in the design of the study, in the collection, analysis, and interpretation of data or in writing the manuscript.
Author details {5a}	Morrow A^1^, Gray SR^1^, Bayes HK^3^, Sykes R^1^, McGarry E^3^, Anderson D^3^, Boiskin D^2^, Burke C^2^, Cleland JGF^4^, Goodyear C^5^, Ibbotson T^6^, Lang CC^7^, McConnachie^4^, Mair F^6^, Mangion K^1^, Patel M^8^, Sattar N^1^, Taggart D^9^, Taylor R^6^, Dawkes S^10^, Berry C^1^ ^1^ Institute of Cardiovascular and Medical Sciences, University of Glasgow ^2^ Queen Elizabeth University Hospital, NHS Greater Glasgow and Clyde ^3^ Glasgow Royal Infirmary, NHS Greater Glasgow and Clyde ^4^ Robertson Centre for Biostatistics, Institute of Health and Wellbeing, University of Glasgow ^5^ Institute of Inflammation, Infection and Immunity, University of Glasgow ^6^ General Practice and Primary Care, Institute of Health and Wellbeing, University of Glasgow ^7^ School of Medicine, University of Dundee ^8^ University Hospital Wishaw, NHS Lanarkshire ^9^ NHS Project Management Unit, NHS Greater Glasgow and Clyde ^10^ School for Nursing Midwifery and Paramedic Practice, Robert Gordon University, Aberdeen Concept: C Berry, N Sattar, SR Gray Design: Stuart R Gray, Bayes HK, Anderson D, Cleland JGF, Goodyear C, Lang C, McConnachie, Mair F, Mangion K, Morrow A, Sattar N, Patel M, Sykes R, McGarry E, Taggart D, Taylor R, Dawkes S, Berry C. Steering Group: Bayes H, Taggart D, Dawkes S, Berry C. Patient and Public Involvement: Ibbotson T, Bayes H, Taylor R, Berry C.
Name and contact information for the trial sponsor {5b}	NHS Greater Glasgow & Clyde Clinical Research and Development Ward 11, Level 1 Dykebar Hospital Grahamston Road Paisley, PA2 7DE Contact: Dr Maureen Travers E-mail: maureen.travers@ggc.scot.nhs.uk
Role of sponsor {5c}	The sponsor had no role in the study design; collection, management, analysis, and interpretation of data; writing of the report; and the decision to submit the report for publication, and will have no ultimate authority over any of these activities.

## Introduction

### Background and rationale {6a}

The coronavirus disease-19 (COVID-19) pandemic has had an unprecedented impact on our health and social services, and on the population, society, and the economy [1]. COVID-19 is a systemic illness, and complications include pneumonia and pulmonary thromboembolism [2]. COVID-19 is associated with an increase in cardiovascular risk [2, 3]. Systemic inflammation, direct infection of endothelial cells by severe acute respiratory syndrome coronavirus-2 (SARS-CoV-2) and dysfunction of angiotensin-converting enzyme-2 (ACE2), cause endothelial dysfunction (aka ‘endotheliitis’) [4, 5]. Consequent microvascular dysfunction, thrombosis, and occlusion likely play a key role in multi-organ dysfunction, especially in the lung, heart, and kidney. We have found that myocardial inflammation, occurs in up to one in eight patients after hospitalisation for COVID-19 [6, 7]. Alternatively, impaired myocardial blood flow appears to affect around 1 in 3 patients, and estimated oxygen consumption (VO2max), a measure of aerobic exercise capacity, is persistently reduced during the convalescence phase (1 month) [6]. Coronary and systemic microvascular dysfunction might be an underlying mechanism for persisting exertional breathlessness, chest pain, and fatigue [3–5].

COVID-19 patients may experience a loss of metabolically active tissue, such as skeletal and cardiac muscle [8]. Hospitalisation may be prolonged, and they are at risk of substantial weight loss, including sarcopenia and cachexia [9]. The mechanisms of cachexia are multifactorial and include tissue catabolism secondary to the acute infection, systemic inflammation, cytokine activation, and loss of mobility [10]. These problems may be especially relevant in older patients with COVID-19, and those with pre-existing chronic health problems, of whom there are many. Even a short period of immobilisation, in-hospital or at home, bed rest and the inactivity of sustained quarantine can have clinically relevant effects on muscle and metabolic health. A reduction in physical activity for just 2 weeks can result in significant losses in muscle mass and function [11] and peripheral insulin sensitivity [12]. A systematic review has identified generalised impairments in physical function after COVID-19 [13]. This is important, as muscle mass is a strong predictor of prognosis in many chronic conditions, and frailty, which associates with multimorbidity and socioeconomic deprivation, may be especially relevant.

Pharmacological methods to increase muscle mass are few and the main method to improve muscle mass and function is via resistance exercise. On top of its effects on muscle mass and function, which are not seen with other forms of exercise, resistance exercise is pragmatic in COVID-19 as it can be easily adapted to the patients’ capabilities and performed in patients from the spectrum of bed-bound to ambulatory with the need for little equipment. Resistance exercise has similar broad-ranging health benefits, such as reducing blood pressure and improving glycaemic control [14, 15] and is associated with a reduction in cardiovascular disease (CVD) risk [16]. Importantly for this trial, resistance training during periods of reduced activity can attenuate declines in muscle mass and function [17].

Many patients who have survived COVID-19 describe persisting health problems. The National Institute for Health and Care Excellence (NICE), the Scottish Intercollegiate Guidelines Network (SIGN) and the Royal College of General Practitioners (RCGP) have defined Long COVID as ‘signs and symptoms that develop during or following an infection consistent with COVID-19 which continue for more than 12 weeks and are not explained by an alternative diagnosis’ [16]. Using clinical data from the over four million people who downloaded the COVID Symptom Study app [17] developed by the health science company, ZOE, Spector and colleagues at Kings College London have provided insights into persisting health problems of COVID-19 patients who were not admitted to hospital. Their preliminary app data show that more than 1 in 8 individuals still have problems after one month from the date of diagnosis, around 1 in 20 have persisting symptoms at two months and 1 in 50 have symptoms at 3 months. Given the scale of the COVID-19 pandemic, many thousands of individuals are affected. These Long COVID sufferers reported a range of symptoms, including up to 20 different symptoms beyond those used for the initial diagnosis of acute infection. In addition, the UK-based Post-hospitalisation COVID-19 study (PHOSP-COVID) long-term follow-up study of adults discharged from hospital with COVID-19 has found that only 29% of participants felt fully recovered at 6 months after discharge, while 19% had experienced a health-related change in occupation [18]. These results underline that COVID-19 is a multisystem disease that requires a multidisciplinary approach to treatment. The Cardiac Imaging in SARS-CoV-2 (COVID-19) (CISCO-19) study of post-hospitalised patients found that multisystem involvement correlated with predicted peak oxygen utilisation and persistent impairments of physical and psychological wellbeing at 28–60 days post-discharge, distinct from control individuals with similar age-, sex-, and morbidity characteristics [7].

Since SARS-CoV-2 is a new virus, the initial research focus has been to investigate the acute illness through observational studies and trials of acute therapy. As we learn more about the natural history of COVID-19, many patients (millions worldwide) are left with persisting symptoms such as exercise intolerance, breathlessness, and fatigue [2, 19] that may represent persisting dysfunction of the heart, lungs, peripheral tissues, and their microcirculation. It is also possible that COVID-19 was just an intercurrent event that interacted with and exposed an underlying health problem (e.g. chronic lung disease, or iron deficiency). A post-viral syndrome that might be anticipated to affect some patients, as is the case with other viral illnesses, is also relevant. NICE, SIGN and RCGP have highlighted the paucity of randomised trial evidence to show whether treatment of the convalescent phase after COVID-19, with or without hospitalisation, reduces longer-term disability. We believe there is a knowledge-gap relating to the prevention and early treatment of longer-term health problems post-COVID-19.

### Objective {7}

To determine the effects of a resistance exercise intervention on functional capacity and health status following COVID-19 infection.

### Trial design {8}

**Fig. 1:**
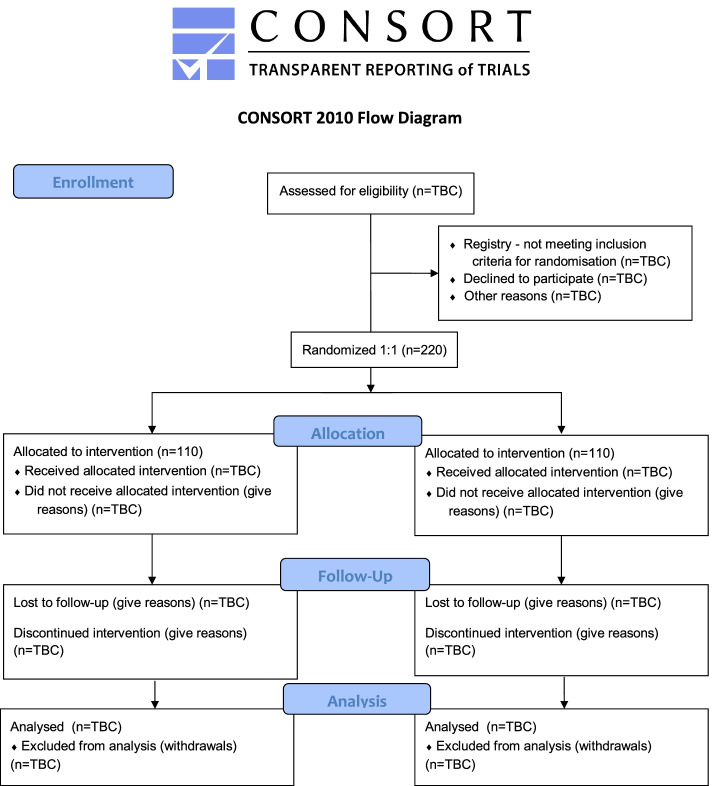
CONSORT diagram

A two-arm parallel randomised, controlled trial with a superiority design comparing usual care alone with usual care plus resistance exercise. Baseline data will be measured prior to randomisation, and follow-up data will be collected 3 months post-randomisation. A CONSORT diagram is presented in Fig. 1.

## Methods: participants, interventions and outcomes

### Study setting {9}

This multi-centre trial includes sites in Glasgow (Queen Elizabeth University Hospital and Glasgow Royal Infirmary) and Dundee (Ninewells Hospital), with NHS Lanarkshire as a patient identification centre.

### Eligibility criteria {10}

Patients will be classified according to clinical presentation:

- Group A, not hospitalised due to COVID but persisting symptoms for at least 4 weeks leading to medical review;
- Group B, Discharged after an admission for COVID and with persistent symptoms for at least 4 weeks; or
- Group C, Convalescing in hospital after an admission for COVID

Groups A and B = target population for treatment of persisting symptoms post-COVID.

Group C = Target population for prevention of Long COVID.

Potential participants will be invited to the research site where eligibility will be determined based on the inclusion and exclusion criteria (Table 1).

**Table 1:** Inclusion and exclusion criteria

In order to be considered eligible for participation in the study they must:	
*Criterion*	*Inclusion*
1	Positive test for COVID-19 (polymerase chain reaction (PCR) or point of care test)
2	Within 6 months of diagnosis
3	Persistent symptoms for at least 4 weeks (Groups A and B only)
4	Presentation type—one of Groups A, B or C
*Criterion*	*Exclusion*
5	In-patient physiotherapy currently part of the standard care post-intensive care unit (ICU)
6	Not expected to be able to walk within 3 months
7	Unable to provide informed consent
8	Unable to comply with the protocol.
9	Known pregnancy

### Who will take informed consent? {26a}

Written consent will be obtained by a member of the study team during a visit to the research site for the baseline assessment, with no study procedures taking place until consent has been obtained. At the baseline visit, a member of the study team will confirm eligibility.

### Additional consent provisions for collection and use of participant data and biological specimens {26b}

Participants consenting for the study will be invited to provide consent for long-term follow-up of their electronic medical records (with no additional patient contact). Consent will be sought to use study data in other ways such as in pooled analyses of anonymised data including for natural history studies, meta-analyses and health outcomes research. This trial involves collecting a blood sample at the follow-up visit at 3 months post-randomisation.

## Interventions

### Explanation for the choice of comparators {6b}

The comparator chosen is usual care as the aim of the study is to investigate the effects of the resistance exercise intervention in addition to usual care. Clinical management of patients following COVID-19 in the NHS will generally align with practice guidelines from the British Thoracic Society and other key stakeholders such as NICE, RCPGP and SIGN. Local practice may vary according to circumstances. In NHS Greater Glasgow and Clyde (NHSGGC) health board, patients who have been admitted with radiological and/or virology confirmed COVID-19 would be invited for a chest X-ray 12 weeks post-discharge and if there are persisting abnormalities on the X-ray, the patient would be invited to further assessment, predominantly via telephone consultation. Options for the evaluation of post-hospitalisation patients with persisting symptoms include provision of an oxygen saturation monitor, a functional assessment of physical capacity, thoracic imaging, pulmonary rehabilitation, and/or an invitation to attend an out-patient clinic according to clinical need and logistical considerations.

### Intervention description {11a}

#### Resistance exercise training

The exercise programme pack is provided to participants in a single document. There are 3 categories within the pack with exercise for those that are (1) bed-bound, (2) up-to-sit and (3) ambulatory, and within each category, the exercises have different levels of difficulty to allow progression or regression, as appropriate.

Participants will be asked to train daily. Patients will be invited to perform the number of repetitions that leads to a validated resistance exercise specific Rating of Perceived Exertion (RPE) of 8–10 [20]. The use of RPE to prescribe and progress resistance exercise has been shown to be as efficacious as other more complex methods but reported to be the most tolerable and enjoyable method [21]. Once patients can reach 10–20 repetitions of each exercise they will be advised to move to the next level within each category and once able to do this for the top level within the category, to move to the next category. Similarly, if patients are not able to manage 5 repetitions of each exercise they will move down a level and, if at the bottom level within a category, down a category. Exercises are as follows:

- *Bed-bound*, lying chest-press, lying row, lying plantar flexion, lying leg press and bridging
- *Up-to-sit*, seated chest-press, seated row, seated lateral raises, seated leg extension, seated plantar flexion, squats
- *Ambulatory*, press-ups, standing lateral raises, seated rows, lunges, calf-raises, squats

A training package will be provided to the site research staff (research nurses, fellows). The pack will be given to patients during an initial face-to-face session where the nurse/therapist will help to select the most suitable category and level of exercise for the patient, and to demonstrate these and ensure patients are comfortable performing the exercises. Every 2 weeks a member of the research team will contact the patients to ensure they are happy with the exercises and to help overcome any issues that have arisen. If the patient is in hospital, the contact may be daily, as needed.

The instructional pack (with video links and pictures) and an exercise log will be provided to empower each patient. The pack was reviewed by our Patient and Public Involvement (PPI) group (4 November 2020) and feedback incorporated to reflect co-design of the programme. Based on our experience in the CISCO-19 study [6], we believe the exercise intervention will be feasible for most patients who have the capacity to follow and adhere to verbal and/or written instructions. We aim for a widely generalisable intervention that can be taken up by most post-COVID-19 patients. The programme is designed to ensure simplicity and safety and remove barriers to implementation, household objects, e.g. a bottle of water and a tin of food, which would be available to all participants, or exercise resistance bands are also an option, according to patient preference.

### Criteria for discontinuing or modifying allocated interventions {11b}

In accordance with the Declaration of Helsinki, participants can withdraw from the trial at any time for any reason. Withdrawal criteria: A participant may withdraw from the study at any time. There are no specific withdrawal criteria, although clinicians can withdraw patients as appropriate and record the reasons. All patients will be followed up for clinical outcomes unless consent is specifically withdrawn.

Pregnancy is an exclusion criterion. If after enrolment the participant becomes pregnant, this is not considered an adverse event (AE) or serious adverse event (SAE) in this trial unless a negative or consequential outcome is recorded for the mother or child/foetus. However, the patient would then be invited to withdraw from the study.

### Strategies to improve adherence to interventions {11c}

Every 2 weeks, a member of the research team will contact the patient to ensure they are doing the exercises, checking there are no ill effects to provide encouragement and motivation and answer questions and help overcome any issues that have arisen. If the patient is in hospital, the contact may be daily, as needed. An exercise log will be provided to empower each patient. This log will not only record adherence to the exercise but also capture any post-exercise issues such as fatigue, dizziness and soreness.

### Relevant concomitant care permitted or prohibited during the trial {11d}

All aspects of clinical care will be permitted, and episodes of care recorded in the electronic case report form (eCRF).

### Provisions for post-trial care {30}

At the end of the trial, participants will be returned to usual care as defined by local and national guidelines at that time.

### Outcomes {12}

#### Primary outcome

Incremental shuttle walk test (ISWT) at 3 months.

The ISWT [20]. This is a validated measure of functional capacity, with good test-retest reliability and evidence of being responsive to rehabilitation interventions. The ISWT is used as a Tier 2 evaluation in the post-hospitalisation COVID-19 (PHOSP-COVID) study [18]. By adopting the ISWT as the primary outcome measure, our study will complement PHOSP-COVID, align with the protocol (for those patients who have been enrolled), and potentially, enhance co-enrolment into this observational study.

Oxygen saturation, heart rate and respiratory rate will be measured at the start and end of the ISWT. They will be exploratory outcomes.

#### Secondary outcomes at 3 months (unless stated otherwise)

1. Spirometry: we will record peak expiatory flow rate, forced vital capacity and forced expiratory volume in 1 s.
2. Handgrip strength: we will measure handgrip strength using a Jamar dynamometer 3 times in the dominant hand.
3. Short Physical Performance Battery (SPPB): we will record the time taken to walk 4 m, time to perform 5 chair rises, and side-by-side, semi-tandem and tandem stands balanced stands with participants asked to hold each for 10 seconds [22]
4. Euroqol-5 dimension (EQ5D) [23]
5. Patient health questionnaire-4 (PHQ) [24]
6. Illness perception (Brief IPQ) [25]
7. Duke Activity Status Index (DASI) [26]
8. International Physical Activity Questionnaire (IPAQ-SF) short-form [27]
9. Fatigue severity scale [28]
10. Medical Research Council (MRC) dyspnoea score [29]
11. Frailty assessed using 1) Fried frailty phenotype: five criteria: weight loss; exhaustion; grip strength; low physical activity; and slow walking pace [30]; 2) Clinical Frailty Scale [31]
12. Episodes of care will be recorded with the following recorded based on the previous 3 months: number of visits/appointments and average duration of contact with GP, GP practice nurses, community assessment centre, physiotherapy, rehabilitation service, accident and emergency, acute medical receiving unit, coronary care unit, ICU, medicine, care of elderly and surgery.
13. Hospitalisation for any reason over the previous 3 months will be recorded.
14. At 1 and 10 years we will also use E-linkage to hospital records to record episodes of care and death (and their causes) and medication use.

The secondary outcomes are intended to align with the post-hospitalisation COVID-19 study (PHOSP-COVID; ISRCTN10980107) assessments at 3-months.

#### Exploratory outcomes at 3 months

1. Exercise dose achieved (1) daily log, (2) level attained and within-subject change from baseline
2. Post-exertional malaise questionnaire [32]
3. Vital parameters of cardio-respiratory function, e.g. oxygen saturation, heart rate, respiratory rate at baseline and during follow-up
4. Accelerometery to measure time spent in light, moderate and vigorous physical activity [33] (Glasgow site)
5. Biobank tissues samples for future biomedical research
6. Observational sub-study of small vessel function using myography in arterioles isolated from gluteal skin biopsies and laboratory analysis of adipose tissue, cells and molecules from the biopsy

Research blood samples: Exploratory analyses may include - Haemostasis - Claus Fibrinogen, D-Dimer, factor VIII (one stage), von Willebrand factor (VWF) antigen, VWF:glycoprotein-1ba, Antithrombin activity, Protein C activity, Free protein S; Cardiac - N-terminal pro-brain natriuretic peptide, hs-troponin; Vascular cytokines – endothelin-1, intracellular adhesion molecule, vascular cell adhesion molecule, p-selectin, interleukin-6, VWF, Nicotinamide adenine dinucleotide phosphate oxidase 3; Vascular cells and microparticles; Metabolic status – glycated haemoglobin, lipids; Blood ribonucleic acid (RNA) (Tempus™ or PAXgene®) including for Real Time-PCR of SARS-CoV-2 and RNA for vascular biology; Inflammation – hs-CRP, ST2; Immune response antibodies; Collagen metabolites; Deoxyribonucleic acid (DNA) - The buffy coat will be stored for DNA analysis e.g. candidate gene single nucleotide polymorphisms, other variants. Residual blood will be saved for future analyses of interest.

### Participant timeline {13}

The participant timeline is shown in Table 2.

**Table 2:** Schedule of enrolment, interventions and assessments

Visit	1	2	3
Timeline	Day 1	3 months (+6weeks)	E-linkage
Trial Activity Setting	Enrolment Hospitalised, non-hospitalised COVID-19	Outcome evaluation CRF	1 and 10 years
Screening – inclusion/exclusion criteria including DASI questionnaire	√		
Written informed consent	√		
General health status check	√		
Medical history/clinical status^2^	√	√	
Vital signs (heart rate, rhythm, BP, height, weight, waist circumference, oxygen saturation, at baseline and during follow-up)	√	√	
Cardiovascular risk factors, risk score	√	√	
Routine blood samples as per standard of care	√	√	
Spirometry	√	√	
Handgrip strength	√	√	
Short Physical Performance Battery (SPPB)	√	√	
Accelerometer (Glasgow only)	√	√	
PROMS including EQ5D, PHQ, Illness perception (Brief IPQ), Duke Activity Status Index (DASI), International Physical Activity Questionnaire (IPAQ-SF) short-form, Fatigue questionnaires	√	√	
Frailty 1) Fried 5-criteria phenotype, 2) Clinical Frailty Scale	√	√	
Training - Hospital, CRF, on-line, telephone			
Randomisation	√		
Training (resistance exercise)	√		
Incremental shuttle walk test (ISWT)	√	√	
Exercise dose (log, level, adherence)	√	√	
Episodes of care (primary, secondary, physiotherapy, rehabilitation)		√	√
Research blood sample		√	
Gluteal Biopsy (optional vascular biology sub-study)		√	
Data collection – clinical endpoints (collected via hospital Portal/Trakcare systems) and e-record linkage			√

### Sample size {14}

The minimum clinically important between-group difference in the ISWT at follow-up (3 months) = 46 m, SD=105, sample size for 80% power, 5% significance, no loss to follow-up (LTFU) = a minimum of 85/group; allowing for LTFU and incomplete data, the sample size is *n*=110/group (*n*=220 total).

### Recruitment {15}

All patients presenting at participating acute hospitals in Glasgow and Dundee and Primary Care settings, e.g. General Practice, community assessment centres, Community Health Centres or Community Test Centres, with COVID-19 can be considered for inclusion. A screening log with the reasons for not being enrolled will be prospectively completed.

We estimate recruiting participants from existing networks (e.g. Long COVID Scotland groups) from primary care. Recruitment from General Practice will be facilitated by providing information about the study i.e. poster, patient information sheet, consent form, and NRS Primary Care Network using: electronic records search; mail/text invitations; alerts for prospective/opportunistic identification.

Healthcare staff who participate in this study will adopt a consecutive approach to enrolment in order to minimise selection bias and provide a study cohort that is representative of patients treated during usual care. The results are intended to have external relevance and be transferable to clinical practice. This approach will also facilitate timely delivery of the trial.

## Assignment of interventions: allocation

### Sequence generation {16a}

Participants will be allocated to usual care or usual care plus a resistance exercise, using a mixed minimisation/randomisation procedure. Within each study site, out of every ten participants, 8 will be allocated according to a minimisation algorithm, designed to maintain balance with respect to study site, age (<40, 40–49, 50–59, 60–69, or ≥70 years), sex, clinical presentation group (A, B, or C), and history of COVID-19 pneumonia. The remaining two patients in each block of ten will be allocated at random. The sequence in which patients will be allocated by minimisation or randomisation will be computer-generated in advance and known only to those responsible for maintaining the allocation system.

### Concealment mechanism {16b}

Allocations will be performed via a study-specific web portal, at the end of the enrolment visit, after collection of baseline data. The research nurse requesting each allocation will not know which group the patient will be allocated to in advance.

### Implementation {16c}

Participants are enrolled on the randomisation system by research nurses who are automatically informed of the intervention allocation by email.

## Assignment of interventions: Blinding

### Who will be blinded {17a}

The trial statisticians will be blind to allocation. As this is an exercise intervention blinding of the participants or research staff, who will instruct the participants in the intervention, is not possible.

### Procedure for unblinding if needed {17b}

Not applicable—the design is open label with only the trial statistician being blinded so unblinding will not occur.

## Data collection and management

### Plans for assessment and collection of outcomes {18a}

Data management will follow the processes of the Glasgow Clinical Trials Unit: http://glasgowclinicaltrials.org/sops/. Data will be collected via a study-specific electronic case report form (eCRF), developed by the Robertson Centre for Biostatics (RCB), University of Glasgow (within the Glasgow Clinical Trials Unit). The eCRF has built-in logic steps and point of entry validation for selected fields. RCB data management staff will run validation checks on the data during the trial, according to a pre-specified Data Management Plan. RCB statistical staff will also raise data issues that arise as part of the statistical programming. The database will only be locked for the final analysis after all data management and statistical data validation checks have been satisfactorily resolved.

### Plans to promote participant retention and complete follow-up {18b}

No further strategies, than those described to maintain engagement with intervention, are planned.

### Data management {19}

All data handling procedures will be detailed in a study-specific data management plan. All quantitative data collected as part of the study will be securely transferred to the Robertson Centre for Biostatistics Clinical Trials Unit (CTU) for data entry and checking in accordance with their standard operating procedures (SOPs).

Data will be validated at regular intervals during the study. Data discrepancies will be flagged to the study site, and any data changes will be recorded to maintain a complete audit trail (reason for change, date change made, who made change).

### Confidentiality {27}

Access to the collated participants’ data will be restricted to the principal investigator and appropriate research study staff as required. All laboratory samples, completed forms, reports and other records will be identified using a unique participant ID number to maintain participant confidentiality.

### Plans for collection, laboratory evaluation and storage of biological specimens for genetic or molecular analysis in this trial/future use {33}

Blood is collected at 3 months according to a sample handling manual approved by the sponsor. The plasma is stored at -80°C in a secure biorepository for analyses that will be specified and approved in a future ethics amendment.

## Statistical methods

### Statistical methods for primary and secondary outcomes {20a}

This study will have a comprehensive Statistical Analysis Plan, which will govern all statistical aspects of the study, and will be authored by the Trial Statistician database lock and unblinding of the statistical team. The Statistical Analysis Plan (SAP) will be based on intention to treat principles in line with Consolidated Standards of Reporting Trials (CONSORT) guidelines. The analysis will focus on estimation of treatment effect differences with 95% CIs and p-values. All pre-specified secondary outcome analyses will be reported in study publications.

Primary efficacy analysis. Alternative hypothesis: In patients with persisting symptoms during the convalescence phase after COVID-19, resistance exercise increases exercise capacity measured by the incremental shuttle walk test. The null hypothesis reflects no difference in exercise capacity between the groups.

The primary outcome (ISWT distance at 3 months) will be analysed using a linear regression model, with randomised group, baseline ISWT distance, age, sex, clinical presentation group, and history of COVID-19 pneumonia as covariates. Similar methods will be applied to secondary outcomes, using linear, binary logistic, or ordinal logistic regression methods, as appropriate.

### Interim analyses {21b}

No interim analysis is planned in the current study.

### Methods for additional analyses (e.g. subgroup analyses) {20b}

Analyses of the primary outcome will be done in relation to subgrouping variables including age, sex, clinical presentation group, and history of COVID-19 pneumonia. This will involve extending the primary analysis regression model to include interaction terms. Complier Average Causal Effects analysis will be employed to estimate the effect of the exercise intervention amongst those who are able to comply with it.

### Methods in analysis to handle protocol non-adherence and any statistical methods to handle missing data {20c}

Participants found to be ineligible for the study, who were randomised in error, will be excluded from all analyses. Other forms of non-compliance that can be identified from the study data (e.g. visits outside of pre-specified windows) will be reviewed (blind to group allocation) and classified as major or minor. Similarly, any other protocol non-compliances will be assessed prior to locking the database and classified as major or minor. Sensitivity analyses will be done after the exclusion of participants with major protocol non-compliances.

Missing data will not be imputed for main primary and secondary analyses. As sensitivity analyses, these analyses will be repeated, using multiple imputation with chained equations for missing data.

### Plans to give access to the full protocol, participant level-data and statistical code {31c}

The data sets and codes to be used for these analyses will be available from the corresponding author on reasonable request.

## Oversight and monitoring

### Composition of the coordinating centre and trial steering committee {5d}

The Trial Steering Committee comprises:

- Prof Susan Dawkes (CHAIR)
- Prof Sally Singh (Independent member)
- Dr Hannah Bayes (Non-Independent member)
- Prof Colin Berry (Chief Investigator)
- Prof Stuart Gray (Non-Independent member)
- Dr Tracy Ibbotson (Independent member)
- Prof Alex McConnachie (Study Statistician)
- Dr Maureen Travers (Sponsor representative)
- Ms Diann Taggart (Study Project Manager/TSC facilitator)

### Composition of the data monitoring committee, its role and reporting structure {21a}

Since the intervention is low-risk and the trial is a non-clinical trial of an investigational medicinal product the sponsor has deemed that a data monitoring committee is not needed.

### Adverse event reporting and harms {22}

Where an SAE requires recording; full details including the nature of the event, start and stop dates, severity, relationship to research product and/or trial procedures, and the outcome of the event will be recorded in the patient’s medical notes and CRFs. These events will be monitored and followed up until satisfactory resolution and stabilisation. All SAEs will be recorded in the eCRF within 24 hours of becoming aware of the event and provide further follow-up information as soon as available. The Site Principal Investigator (PI) will use medical judgement in assigning seriousness, causality, severity and expectedness with reference to the trial protocol and Reference Safety Information. Relatedness and expectedness will be determined by the site team and site principal investigator. The sponsor will verify data collection and SAEs, according to the trial protocol. The sponsor will reporting safety information to the Chief Investigator or delegate for the ongoing assessment of the risk / benefit and in collaboration with the Chief Investigator the annual safety report to the Research Ethics Committee and until the End of the Study, at which time an End of Study form will be submitted. The Trial Steering Committee will review safety in accordance with a predefined charter, periodically reviewing recruitment and the overall progress of the trial and liaising with the sponsor regarding safety issues.

A Related Unexpected Serious Adverse Event (RUSAE) will be reported to the sponsor who in turn will report the RUSAE to the ethics committee. The study Data Centre will provide an eCRF for central data collection of AEs and SAEs and they will be reported to the Trial Steering Committee which is scheduled to meet on a 6-monthly basis until the end of the study.

### Frequency and plans for auditing trial conduct {23}

There is no separate monitoring plan for the study. An audit of all entries into the eCRF was deemed to be a sufficient monitoring plan for a non-Clinical Trial of an Investigational Medicinal Product (CTIMP).

### Plans for communicating important protocol amendments to relevant parties (e.g. trial participants, ethical committees) {25}

Any amendments to the protocol will be approved by the local ethics committee prior to implementation and the ClinicaTrials.gov registration will be updated. All investigators and patients enrolled in the trial will be informed.

Any deviations from the Protocol will be fully documented using a report form.

### Dissemination plans {31a}

The study findings will be disseminated primarily via conference presentations, scientific papers, social media and public engagement activities. All authors read and approved the final manuscript.

## Discussion

The current study is a multi-centre, two-arm randomised controlled trial. It has been designed to assess the effect of a resistance exercise intervention on functional capacity and health status following COVID-19 infection. With many patients suffering from the deleterious effects of COVID-19 infection investigating potential prevention and treatment interventions is of the utmost importance.

The current study builds on previous work which has investigated the effects of exercise training on exercise capacity and will compliment ongoing work. For example, it has been shown that a short (6 weeks) supervised rehabilitation programme, involving two supervised sessions of aerobic and resistance exercise per week, in people (*n*=32) with persisting symptoms (physical and/or psychological) post-covid was feasible, with no dropouts due to worsening symptoms. On top of this, there were significant improvements in ISWT performance and symptoms of fatigue, cognition and respiratory symptoms [34]. The majority of other ongoing studies in trial registers are focused on respiratory or aerobic exercise. The current study will extend these early findings in a larger cohort and will focus on resistance exercise only, allowing us to delineate the effects of different forms of exercise and optimise rehabilitation.

Whilst it is promising that previous work has reported that exercise training did not result in worsening symptoms, this was in a small number of participants and so remains something that we are cognizant of in this trial. Whilst we are cautious about making comparisons between myalgic encephalomyelitis or chronic fatigue syndrome (ME/CFS) and the persisting symptoms post-COVID, some symptoms such as post-exertional malaise (PEM) do occur in people with persisting symptoms post-COVID. Indeed, this was highlighted during our Patient and Public Involvement meetings in the study design phase, specifically that an intervention following the principles of graded exercise therapy (GET) may result in harm, rather than benefit, to participants. However, the current study does not follow the GET model, and the exercise intervention is different. The current study involves simple resistance exercises, with no aerobic exercise as part of the intervention. The exercise prescription is based on participants’ perception of effort and so the intensity and volume of exercise will vary daily, empowering the participant to adapt the level of activity based on how they feel and not to meet prescribed targets. Progression, or indeed regression, within the intervention also works on the same principle. On top of this, we are asking participants to record any post-exercise symptoms and discuss these with them during regular telephone calls and adapt the intervention accordingly.

In conclusion, the current study is investigating the effects of a novel resistance exercise therapy for the prevention and early treatment of the long-term physical effects of COVID-19 and will further our understanding of optimal post-COVID care for patients.

## Trial status

The study began recruitment in 12 May 2021 and will be ongoing until the last patient’s last visit (anticipated Q3, 2023). Current protocol: version 2 (02.04.2022).

## Supplementary Information

**Additional file 1.** Patient Informed Consent Form – Main Study.

## Data Availability

Any data required to support the protocol can be supplied on request.

## Abbreviations

ISWT: Incremental shuttle walks test
SPPB: Short performance physical battery
NHS: National Health Service
REC: Research Ethics
ACE2: Severe angiotensin-converting enzyme-2
SARS-CoV-2: Acute respiratory syndrome coronavirus-2
COVID-19: Coronavirus disease-19
CVD: Cardiovascular disease
NICE: National Institute for Health and Care Excellence
SIGN: Scottish Intercollegiate Guidelines Network
RCGP: Royal College of General Practitioners
CISCO-19: Cardiac Imaging in SARS-CoV-2 (COVID-19)
PCR: Polymerase chain reaction
ICU: Intensive care unit
NHSGGC: NHS Greater Glasgow and Clyde
RPE: Rating of Perceived Exertion
PPI: Patient and Public Involvement
AE: Adverse event
SAE: Serious adverse event
eCRF: Electronic case report form
PHOSP-COVID: Post-hospitalisation COVID-19
Brief IPQ: Illness perception
DASI: Duke Activity Status Index
IPAQ-SF: International Physical Activity Questionnaire
EQ5D: Euroqol-5 dimension
MRC: Medical Research Council
CTU: Clinical Trials Unit
SOPs: Standard operating procedures
SAP: Statistical Analysis Plan
CONSORT: Consolidated Standards of Reporting Trials
PI: Principal Investigator
RUSAE: Related Unexpected Serious Adverse Event
CTIMP: Clinical Trial of an Investigational Medicinal Product
PEM: Post-exertional malaise
GET: Graded exercise therapy
ME/CFS: Myalgic encephalomyelitis or chronic fatigue syndrome
VWF: Von Willebrand factor
RNA: Ribonucleic acid
DNA: Deoxyribonucleic acid
RCB: Robertson Centre for Biostatics
