# Supplementary material for: Prevention and early treatment of the long-term physical effects of COVID-19 in adults: design of a randomised controlled trial of resistance exercise—CISCO-21
Source: Trials. 2022 Aug 15;23:660. doi: 10.1186/s13063-022-06632-y (PMC9376905; doi:10.1186/s13063-022-06632-y)
Supplement: Supplementary file 1 — Additional file 1. Patient Informed Consent Form – Main Study. [file 13063_2022_6632_MOESM1_ESM.docx]

| **PATIENT INFORMED CONSENT FORM – MAIN STUDY**  Prevention and early treatment of the long-term effects of COVID-19: a randomised clinical trial of resistance exercise. | |
| --- | --- |
| **PI/Site Name:** | **Participant Trial Number:** |
| **Please Initial boxes** | |

| 1. | I confirm that I have read and understood the patient information sheet version 1.1, dated dd/mm/yyyy, and that I have had any questions I have about the study answered. | | | |  | |
| --- | --- | --- | --- | --- | --- | --- |
| 2. | I understand that my participation is voluntary and that I am free to withdraw at any time, without giving any reason, and without my medical care being affected. | | | |  | |
| 3. | I understand that relevant sections of my medical notes and data collected during the study may be looked at by the study team, responsible individuals from the Sponsor, NHS Greater Glasgow and Clyde, and from regulatory authorities where it is relevant to my taking part in this study. I give permission for these individuals to have access to my records. | | | |  | |
| 4. | I agree that my General Practitioner (GP) will be informed of my participation. | | | |  | |
| 5. | I agree that my contact details i.e. name, address, postcode, telephone numbers, can be retained by the study team for use in relation to study procedures. | | | |  | |
| 6. | I agree to my pseudonymised data being held on servers located within and out with the UK for research purposes. Access to data will be managed by the Co-sponsors in line with Data Protection Legislation. I understand that this form will be uploaded to a secure database in the University of Glasgow. | | | |  | |
| 7. | I agree to long term follow-up information by record linkage being collected on my future wellbeing and treatment from NHS and Government health records, and that my identifiable information will be retained for this purpose. | | | |  | |
| 8. | I agree to take part in this study | | | |  | |
| 9. | I agree to a DNA and RNA blood sample being taken for use in future research. | | |  | |  |
|  |  | | |  | |  |
|  | **Optional** | | | YES | | NO |
| 10. | I agree for you to keep my personal details to allow me to be contacted regarding future ethically approved studies. | | |  | |  |
| 11. | I agree that anonymised surplus blood/DNA/RNA samples may be retained for use in ethically approved future research. | | |  | |  |
| 12. | I agree to wear the watch (accelerometer) to measure my body movement. | | |  | |  |
|  |  | | |  | |  |
| ______________________________ | | ______________________________ | ________________________ | | | |
| **Patient Name (Print)** | | **Patient Signature** | **Date** | | | |
|  | |  |  | | | |
| _____________________________ | | _____________________________ | ________________________ | | | |
| **Person Obtaining Consent (Print)** | | **Signature** | **Date** | | | |
| 3 copies - one for patient, one for notes, and one for study site file | | | | | | |
